# Puerarin Suppress Apoptosis of Human Osteoblasts via ERK Signaling Pathway

**DOI:** 10.1155/2013/786574

**Published:** 2013-06-12

**Authors:** Ling-juan Liu, Li-qun Liu, Tao Bo, Shi-jun Li, Zhen Zhu, Rong-rong Cui, Ding-an Mao

**Affiliations:** ^1^Department of Pediatrics, The Second Xiang-Ya Hospital, Central South University, 139 Middle Renmin Road, Changsha, Hunan 410011, China; ^2^Institute of Metabolism and Endocrinology, The Second Xiang-Ya Hospital, Central South University, 139 Middle Renmin Road, Changsha, Hunan 410011, China

## Abstract

Puerarin, the main isoflavone glycoside extracted from Radix Puerariae, is an isoflavone traditional Chinese herb. Previous studies have demonstrated that puerarin could regulate osteoblast proliferation and differentiation to promote bone formation. However, the effect of puerarin on the process of human osteoblasts (hOBs) apoptosis is still unclear. In this study, we detected the function of puerarin on serum-free-induced cell apoptosis using ELISA and TUNEL arrays and then found that the mortality of hOBs was significantly decreased after exposure to 10^−10^–10^−6^ M puerarin and reached the maximal antiapoptotic effect at the concentration of 10^−8^ M. In addition, compared with the control group, puerarin notably increased the Bcl-2 protein levels while it decreased the Bax protein levels in the hOBs in a dose-dependent way. 10^−7^ M puerarin decreased the Bax/Bcl-2 ratio with a maximal decrease to 0.08. Moreover, puerarin activated ERK signaling pathways in hOBs, and the antiapoptotic effect induced by puerarin was abolished by incubation of ERK inhibitor PD98059. Similarly, the estrogen receptor antagonist ICI182780 also suppressed the inhibitory effect of puerarin on hOBs apoptosis. In conclusion, puerarin could prevent hOBs apoptosis via ERK signaling pathway, which might be effective in providing protection against bone loss and bone remolding associated with osteoporosis.

## 1. Introduction

Osteoblasts, mononuclear specialized cells derived from mesenchymal precursor cells, are responsible for bone formation, deposition, and mineralization [1, 2], playing an essential role in the maintenance of the stability of bone microarchitecture. Osteoblast apoptosis, induced by various pathological and physiological factors (e.g., estrogen loss, glucocorticoids, weightlessness, and aging), breeds a series of bone disorders. Osteoporosis is the most prevalent bone disorder affecting the elders, which is characterized by an imbalance between bone formation and bone resorption [3–5]. Several studies have showed that apoptosis might be the third most common cause of osteoporosis, and 60–80% of osteoblasts were estimated to originally assembled at the resorption pit die by apoptosis [6]. Therefore, apoptosis is generally served as the pivotal target for prevention and/or ameliorate osteoporosis.

Puerarin, 7-hydroxy-3-(4-hydroxyphenyl)-1-benzopyran-4-one 8-*β*-D-glucopyranoside (C_12_H_20_C_9_), is one of the major isoflavonoid compounds extracted from the root of a wild leguminous creeper [7, 8]. It possesses estrogen-like structure and moderates estrogenic activity. As a famous phytoestrogen, current studies have established that puerarin provides a strong protection against osteoporosis through facilitating osteoblast proliferation and differentiation [9, 10]. Although it acts as an important regulatory factor for cell death, the role of puerarin on osteoblast apoptosis and its underlying mechanism of action are still unclear. As we know osteoblasts apoptosis is an extremely complicated event; a series of proteins and signaling pathways (e.g., Bcl-2 family proteins, ERK, MAPK, APJ/PI3-K/Akt, JAK2, and Fas) are reported to be involved in this process in vitro and in vivo [11–15]. In present study, we aim to detect the impact of puerarin on the serum-deprivation-induced hOBs apoptosis along with observing the expression of Bcl-2 and Bax protein using western blotting and the cell signal pathway involved.

## 2. Materials and Methods

### 2.1. Reagents

Puerarin and 17*β*-estradiol were purchased from Sigma, Inc. (USA). *β*-actin, anti-Bcl-2, Bax, ERK, p-ERK antibodies, anti-mouse, and -rabbit IgG peroxidase conjugate antibodies were purchased from Santa Cruz Biotechnology Inc. (Waltham, MA, USA). ICI 182780 and PD98059 were purchased from Calbiochem Corp. (San Diego, CA, USA).

### 2.2. Cell Cultures

hOBs were obtained from traffic accident victims suffering surgery, and this procedure was approved by the local research ethics committee as previously mentioned [16]. Cells isolated from femur were cultured in a-MEM medium (Gibico-BRL. Corp., NY, USA) containing 15% fetal bovine serum, 100 U/mL penicillin, and 100 *μ*g/mL streptomycin at 37°C in a 5% CO_2_ atmosphere. Cells were identified as osteoblasts by the expression of ALP, collagen type I and osteocalcin, and formation of mineralized nodules as lately described [12].

To determine the effect of puerarin on apoptosis of hOBs, the cells were seeded for 24 h followed by culturing for 24 h in serum-free medium, then treated with 0, 10^−9^ M, 10^−8^ M, 10^−7^ M puerarin, or 17 *β*-estradiol (E2, 10 *μ*M) for 48 h. To analyze the effect of ERK or estrogen receptor (ER) inhibition on hOB apoptosis, hOBs were pretreated with 10 *μ*M ERK inhibitor PD98059 or ER antagonist ICI 182780 for 3 h anterior to incubation with 10^−8^ M puerarin for 48 h.

### 2.3. Western Blot Analysis

Immunoblotting was carried out as before [17]. Total protein was extracted with RIPA lysis buffer (Beyotime, China), then protein concentration determined by a Bradford assay, and equal amounts of protein were loaded onto SDS-PAGE and then transferred to PVDF membranes (Invitrogen, Carlsbad, CA, USA). Thereafter, the membranes were blocked with 5% nonfat milk in PBS for 1 h at room temperature and then incubated with anti-Bcl-2, anti-Bax (1 : 500), or anti-*β*-actin (1 : 1000) antibodies overnight at 4°C. Resultant protein bands after incubation with a proper secondary antibody were visualized by chemiluminescence. The absorbance values of target proteins were analyzed through Gel-Pro 4.0 gel image analysis software. The absorbance ratio of each protein to internal reference was represented as the relative amount of target proteins [18].

### 2.4. Measurement of ERK Activation

hOBs were treated with 10^−8^ M puerarin for 0–45 min. The cell layers were washed twice with cold PBS and then lysed with a buffer consisting of 20 mM Tris-HCl (pH 7.5), 150 mM NaCl, 1% Triton X-100, 10 mM NaH_2_PO_4_, 10% glycerol, 2 mM Na_3_VO_4_, 10 mM NaF, 1 mM ABSF, 10 *μ*g/mL leupeptin, and 10 *μ*g/mL aprotinin. Western blot analysis was carried out as previously described [19, 20]. Equal amount of proteins were transferred onto PVDF membranes, then incubated with anti-ERK or anti-p-ERK monoclonal antibodies (1 : 500). The ECL detection kit was used for detection.

### 2.5. Measurement of Cell Apoptosis

#### 2.5.1. Cell Death ELISA Detection

Cell death ELISA detection was performed for detecting the apoptosis levels as previously described [21]. Cell death detection ELISA kit (Roche Diagnostics GmbH, Roche Molecular Biochemicals, Mannheim, Germany) was used for detection, according to the kit protocol. Briefly, cells were plated at a density of 10,000 cells/well in 24-well plates for 1 day followed by culture in serum-free medium for 48 h in the absence or presence of 0–10^−6^ M puerarin or 10 *μ*M E2. Cells were rinsed with PBS and incubated with 0.5 mL of lysis buffer at 4°C for 30 min, then centrifuged for 10 min at 15,000 rpm. Aliquots of the supernatant were tested for the rate of apoptosis through the cell death detection kit.

#### 2.5.2. TUNEL Assay

Terminal deoxynucleotidyl transferase-mediated deoxyribonucleotide triphosphate nick end-labeling (TUNEL) was generally used to assess cell death. hOBs were washed with PBS after cultured in serum-deprivation medium for 48 h in the absence or presence of 10^−8^ M puerarin and then fixed with 4% paraformaldehyde for 30 min at room temperature. Thereafter, the cells were incubated with the TUNEL reaction mixture(Roche Molecular Biochemicals, Indianapolis, IN) for 60 min at 37°C followed by labeling with fluorescein isothio cyanate (FITC)-conjugated anti-fluorescein anti-goat antibody (Fab fragment) for an additional 30 min. The nuclei were counterstained with 4,6-diamidino-2-phenylindole (DAPI). Finally, TUNEL-positive cells were photographed on an Olympus microscope.

### 2.6. Statistical Analysis

The data are presented as mean ± standard deviation (SD). Statistical analyses of the data were performed through one-way analysis of variance (ANOVA) and the LSD post hoc test for multiple comparisons. *P* < 0.05 was considered the statistical significant difference. All experiments were repeated at least three times.

## 3. Results

### 3.1. Puerarin Protects the hOBs from Serum-Free-Induced Apoptosis

**Figure 1:**
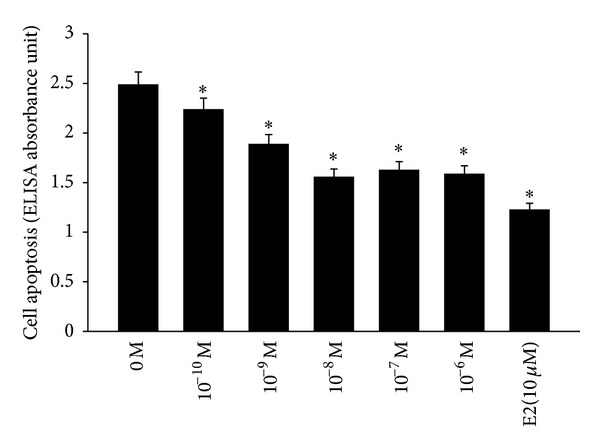
Puerarin protected the hOBs from serum-free-induced apoptosis. Cells were treated with 0–10^−6^ M puerarin, and cells exposed to 10 *μ*M E2 served as the positive control. Apoptosis were measured by ELISA according to the kit specification. The bars represent the mean ± SD (*n* = 6), *P < 0.05, as compared with the control cells.

**Figure 2:**
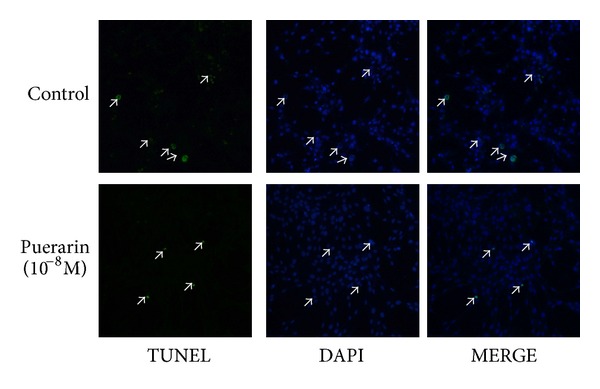
Effect of puerarin on apoptosis of hOBs measured by TUNEL assay. Cells were exposed to 10^−8^ M puerarin for 48 h, while the control group was incubated with serum-free medium for 48 h. Apoptotic nuclei were detected by TdT-mediated dUTP nick end-labeling (TUNEL). Arrows show apoptotic cells. Original magnification ×100.

Our study used ELISA assays to assess hOBs apoptosis cultured in serum-depravation medium for 48 h in the presence of 0–10^−6^ M puerarin or 10 *μ*M E2. Results showed that after exposure to puerarin the apoptotic cells at 10^−10^ M (2.23 ± 0.14 ELISA absorbance units), 10^−9^ M (1.89 ± 0.16 ELISA absorbance units), 10^−8^ M (1.54 ± 0.13 ELISA absorbance units), 10^−7^ M (1.62 ± 0.15 ELISA absorbance units), and 10^−6^ M (1.58 ± 0.12 ELISA absorbance units) puerarin were less than those of the negative control group (2.51 ± 0.11 ELISA absorbance units, all *P* < 0.05), while they were higher than the positive group exposed to 10 *μ*M E2 (1.30 ± 0.12 ELISA absorbance units, *P* < 0.05). Puerarin reached the maximal antiapoptotic effect at the concentration of 10^−8^ M (Figure 1). The results of TUNEL assay also indicated that 10^−8^ M puerarin significantly decreases hOBs apoptosis caused by serum deprivation compared to the control (*P* < 0.05, Figure 2).

### 3.2. Effects of Puerarin on the Expression of Bcl-2 and Bax in hOBs

**Figure 3:**
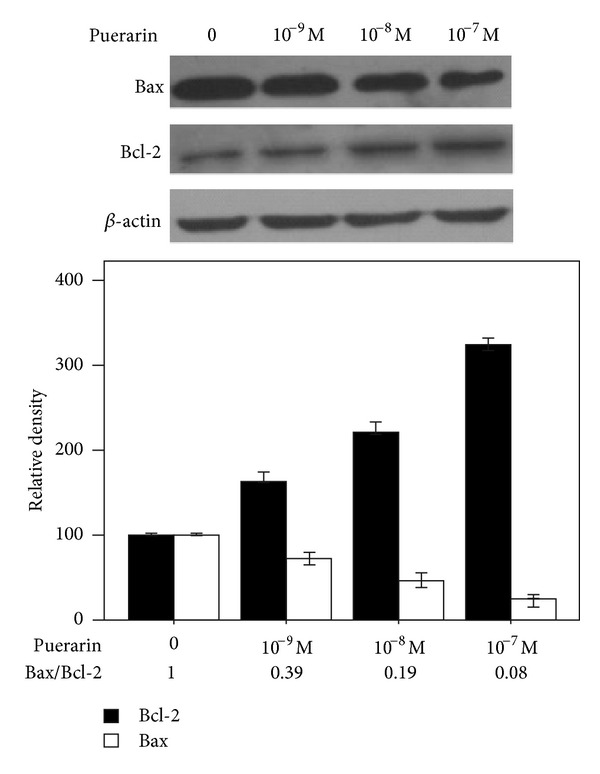
Effects of puerarin on the expression of Bcl-2 and Bax in hOBs. Cells were treated with 0, 10^−9^ M, 10^−8^ M, and 10^−7^ M puerarin for 48 h before collecting proteins. Cell lysates were subjected to western blot analysis and incubated with anti-Bax, anti-Bcl-2, or anti-*β*-actin monoclonal antibodies. Gel-Pro 4.0 gel image analysis software was used to analyze the absorbance values of Bcl-2 and Bax.

Bcl-2 and Bax are the essential members of Bcl-2 family which involved in the process of apoptosis. Western blot analysis was used to detect the expression of Bcl-2 and Bax in hOBs incubated with 0, 10^−9^ M, 10^−8^ M, and 10^−7^ M puerarin. As a result, we found that puerarin increased the levels of Bcl-2 protein, while it decreased the expression of Bax in hOBs in a dose-dependent manner (all *P* < 0.05, Figure 3). The Bax/Bcl-2 ratio was set to 1 in the control group, and 10^−7^ M puerarin could downregulate the Bax/Bcl-2 ratio with a maximal decrease to 0.08 (all *P* < 0.05, Figure 3).

### 3.3. Puerarin Activated ERK Signaling Pathway in hOBs

**Figure 4:**
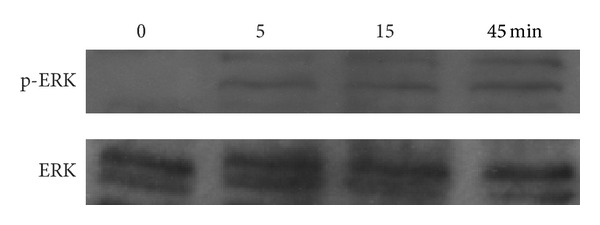
Effects of puerarin on ERK signaling pathways in hOBs. Anti-ERK and anti-p-ERK monoclonal antibodies were used to perform in western blotting. Cells were exposed to 10^−8^ M puerarin for 0–45 min. The levels of ERK and p-ERK were measured by densitometry of autoradiographs.

ERK signaling cascades is a classic pathway involved in the regulation of cell death. According to our results, 10^−8^ M was the optimum concentration for puerarin to produce the best antiapoptotic protection. Therefore, we used western blotting to confirm the effects of 10^−8^ M puerarin on ERK phosphorylation and found that the levels of phosphorylated ERK was upregulated after 5 min incubation with puerarin compared with the control group (*P* < 0.05). Additionally, this effects was time-dependent with the peak activation of ERK at 45 min of incubation (*P* < 0.05, Figure 4).

### 3.4. ERK Signaling Pathway and Estrogen Receptors Mediated the Antiapoptotic Effects of Puerarin in hOBs

**Figure 5:**
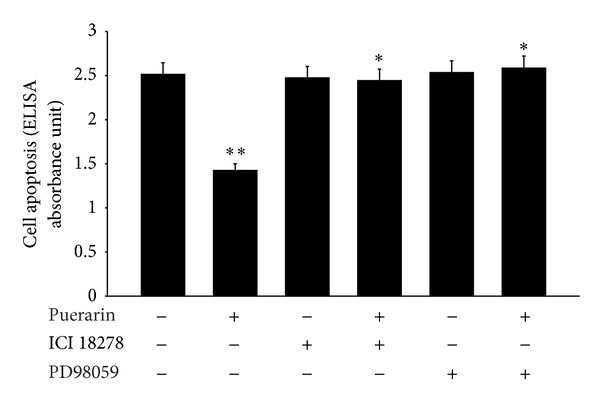
ERK signaling pathway and estrogen receptor mediated the antiapoptotic effects of puerarin in hOBs. Cells were incubated with PD98059 (10 *μ*M) and/or ICI 182780 (10 *μ*M) for 3 h prior to treatment with 10^−8^ M puerarin for 48 h. The bars represented the mean ± SD (*n* = 6). *P < 0.05, as compared with the puerarin-treated cells; **P < 0.05, as compared with the serum-free treated cells.

After detecting that puerarin could activate ERK signaling pathway in hOBs, we further verified whether the puerarin-mediated activation of ERK participates in apoptosis. We examined apoptosis after incubation with 10^−8^ M puerarin and/or PD98059 by ELISA, then detected that the protection effect of puerarin on hOBs was blocked by PD98059, which had little effects on apoptosis alone (*P* < 0.05, Figure 5). Moreover, puerarin was proved to have estrogen-like structure and moderate estrogenic activity, and we found here that ICI 182780 did not affect the process of apoptosis but significantly suppressed the antiapoptotic effect of puerarin on hOBs (*P* < 0.05, Figure 5).

## 4. Discussion

Osteoblast apoptosis is generally regarded as a key component of bone turnover, repair, and regeneration [22]. It is reported that approximately 50–70% of osteoblasts undergo apoptosis during bone regeneration [23]. Puerarin, an isoflavone traditional Chinese herb, is reported to significantly facilitate the survival rate of osteoblasts, and the puerarin-treated rats also displayed a higher rate of bone formation in the osteoblast implants than the control, suggesting that puerarin might regulate osteoblast proliferation and differentiation to promote bone formation in osteoblast implants [24, 25]. In present research, we demonstrated that puerarin alleviates apoptosis of hOBs induced by serum deprivation through activating ERK signaling pathway.

Cell apoptosis is an essential process in maintaining the homeostasis under normal conditions [26]. Members of the Bcl-2 family, including Bcl-2 and Bax, are the main regulators of apoptosis which promote (Bax) or inhibit (Bcl-2) apoptosis [27]. Each of them regulates apoptosis independently [28]. Furthermore, Bcl-2 protein forms heterodimer complexes with Bax proteins, leading to the release of cytochrome C from the mitochondria and induction of cell apoptosis [29]. Previous reporters have proved that puerarin acts on a variety of apoptosis through regulating the expression of Bcl-2 family proteins in vitro, including vascular endothelial cells, vascular smooth muscle cells, and human neurons [30–32]. Here we discovered that puerarin protects hOBs from serum-free-induced apoptosis by upregulating the expression of Bcl-2 and downregulating the expression of Bax and then significantly decreasing the Bax/Bcl-2 ratio in a dose-dependent manner, suggesting that the Bcl-2 family participates in the regulation of the prevention hOBs from apoptosis by puerarin.

To gain further insight into the mechanisms by which puerarin suppresses hOBs apoptosis, we examined ERK signaling pathways. Extracellular signal-regulated kinase (ERK1/2), located at both the cytoplasm and the nucleus of cells, is a multifunctional serine/threonine kinases which induce various of substrates phosphorylation localized in all cellular compartments [33, 34]. It is believed that ERK is involved mainly in the activation of nuclear transcription factors that control cell proliferation, differentiation and apoptosis [35]. Currently, osteoblasts apoptosis induced by serum deprivation was proved to be suppressed significantly through activating the ERK signaling pathway [12, 36]. Present study we investigated the effect of puerarin on ERK and found that puerarin activated ERK phosphorylation and inhibited hOBs apoptosis. This protection was eliminated through pretreatment with PD98059, indicating that ERK signaling pathway was the key link in the antiapoptotic effects of puerarin on hOBs.

Additionally, it is well known that ERs are the major targets for estrogen acting on osteoblasts apoptosis [37]. As a phytoestrogen, puerarin was reported to be closely connected with ERs. In the study of Tiyasatkulkovit et al. [38], ER antagonist ICI182780 substantially inhibited the facilitation of puerarin on osteoblast differentiation. These present findings corroborated that ICI182780 blocked the effects of puerarin on hOBs apoptosis just as was expected, supporting the conclusion that puerarin suppressed hOBs apoptosis in an ER-dependent manner.

## 5. Conclusion

The present study has confirmed the potential benefit of puerarin on hOBs apoptosis in vitro, which is mediated by ERK signaling pathway. It is well known that hOBs apoptosis plays an essential role in the process of osteoporosis. Consequently, we conclude here that puerarin might ameliorate bone loss and promote bone remolding after subjecting to osteoporosis. Further studies are needed to affirm the effect of puerarin on osteoporosis in vivo.
